# Rubber-Stamp-Circular Degrees

**Published:** 1903-06

**Authors:** 


### Rubber-Stamp-Circular Degrees.

And now, for the small sum of $10, the tuft-hunter may have added to his name the imposing degree of doctor of laws, with a handsome parchment, "suitably engrossed," giving him the right to add to his name and his various other titles and degrees, the letters LL.D. This is easy of accomplishment, for the Nashville College of Law, of Nashville, Tenn., is sending out circular letters to physicians offering the degree for sale. The circular is filled in with a typewriter and signed with a rubber stamp, typical of the honor to be conferred on purchasers, and is as follows, being addressed to "Hon. John Brown, M.D." John Brown isn't the name, but it will do as well as any other:

In accordance with a time-honored custom, the college will confer a title upon some worthy educator or jurist of your state, by bestowing the Degree of Doctor of Laws, LL.D., and in harmony with the annual custom established by the leading American and European colleges, after long continued usage, your name has been suggested as one from your state of ability, morality and integrity, and worthy of this distinction, and upon the presentation of the proper application and data, the title will be conferred at the coming meeting of the board of trustees. The desire of the corporation is that this honorary distinction be conferred only upon those who may, in some way, prove an honor to himself, an honor to his country, and an honor to the college. There are no fees attached, as it is purely honorary, except the cost of the issuance of the diploma and engrossing name in same. The college diploma bears a facsimile of the corporate and state seal, is lithographed upon parchment, carefully prepared sheepskin, suitable to frame and place in the home or office, and is a testimonial of honor, prestige, efficiency and worth.

Please fill out the enclosed blank application and return to this office for filing purposes to be kept in the records of the college for future reference, and enclose check for the incidental fee to cover expense of the issuance of the diploma and engrossing your name in same, and it will be placed to your credit upon the books of the college, and if, for any cause, the title be not conferred, the incidental fee will be returned to you by the corporation. However, should you be elected an honorary alumnus of the college, the trustees, the faculty and myself shall be pleased to have you send the institution a large portrait of yourself, properly framed, and suitable to hang on the walls of the college.

We shall take pleasure in sending you a copy of our latest college catalogue, as soon as it comes from the press, containing announcements, official rules and regulations of the college. It sets forth the policy of the college, courses of instruction and full information concerning the institution in a very clear light.

Extending to you personally, and on behalf of the college, our earnest congratulations as a coworker in the domain of the great profession, believe me, my dear sir,

Faithfully yours,
WM. FARR, Dean.

P.S.—The May meeting of the board of trustees will be held the latter part of the present month, and your record of data, etc., should be received in advance of the meeting. You will be duly notified of the action of the board, and if elected to a degree, your diploma will be expressed to you, otherwise, check returned.—R.S.V.P.

The blank referred to for filing purposes is ludicrous. Here are the questions a candidate for the $10 degree must answer as necessary qualifications to his fitness of the $10 honor: "Name in full; P. O. address; age; married or single; nationality; place of birth; profession or avocation; are you a graduate? if so, what course or courses? Where, when and how long did you attend? Have you taken a course by the correspondence method? What degree, if any, do you hold? If so, conferred by what college? When were the degrees conferred? If not a graduate, state time studied in years. When and where admitted to your profession? State experience in years as a member of your profession? What law books, if any have you read? Do you believe in the coeducation of the sexes in a college of law? What public offices if any have you held? What interest do you take in educational affairs? What literary position, if any have you held? Name the church of your choice? What is your political belief in national affairs? Are you a member of any secret society? What? Do you take daily exercise? Do you have good health? Are you the author of any book, thesis or literary article? If so, name them. How much time do you devote daily to study. Was Pope right in saying, drink deep or taste not of the Pierian spring? What magazine, if any, do you read on American legal lore? Incidental fee enclosed, $10."

Just how these questions apply to a candidate's fitness for an honorary degree is hard to say. The JOURNAL has no hesitation in saying, and in fact it has the say-so of the physician who received the circular invitation to buy an honorary degree, that the only qualification he possesses is the fact that he is able to spare the $10 for the "incidental fee." Other than that he has done nothing to warrant the conferring of an honorary degree by a standard college or university. He is not a lawyer, knows nothing about law, save as it applies to medicine he, is not an author of distinction, nor is he an eminent educator. He might come under the $10 rule of the Nashville College of Law by reason of the fact that he takes daily exercise, that he has held literary positions and that he has a leaning toward a certain religion and votes a straight ticket at National elections. But think of the terrible mistake a prospective purchaser might make if, when all swelled up with pride over the distinction of being chosen by so eminent a rubber-stamp institution as the Nashville College of Law for one of its honorary $10 bargain-day degrees, he should answer the Pope query in a manner calculated to chill the heart of the board of trustees. Or possibly he might have grammatic scruples about replying to the question concerning the "coeducation of the sexes" But maybe it would be all right in any event, no matter how poor the answers, if the check "for engrossing" were good. It would be interesting to know how many honorary degrees this institution confers this spring at $10 per.

